# A Comparison of Implementation of Synchronization, Artificial Insemination and Sex-Skewed Semen on Reproductive Performance and Calving Distribution in Beef Herds

**DOI:** 10.3390/ani16101512

**Published:** 2026-05-15

**Authors:** Saulo Menegatti Zoca, George A. Perry, Matthew A. Diersen, Warren C. Rusche, Emmalee J. Northrop-Albrecht, Jerica J. J. Rich, Kaitlin M. Epperson, Stephanie D. Perkins-Oines, Julie A. Walker

**Affiliations:** 1 Department of Animal Science, University of Tennessee, Knoxville, TN 37996, USA; szoca@utk.edu; 2 Department of Animal Science, South Dakota State University, Brookings, SD 57007, USA; 3 Texas A&M AgriLife Research and Extension Center, Overton, TX 75684, USA; george.perry@ag.tamu.edu; 4 Ness School of Management and Economics, South Dakota State University, Brookings, SD 57007, USA; 5 College of Agriculture, Arkansas State University, Jonesboro, AR 72467, USA; 6 College of Agriculture and Natural Sciences, Northwest Missouri State University, Maryville, MO 64468, USA

**Keywords:** artificial insemination, calving distribution, estrous synchronization, fixed-time artificial insemination, reproductive technologies, sexed semen

## Abstract

This study evaluated the implementation of reproductive technologies and their effects on pregnancy rates (PRs) and calving distribution. Producers (*n* = 11) were enrolled in Level 1 or 2 and subsequently transitioned to Level 2 or 3. Level 1: females were exposed to natural service (NS) only versus estrous synchronization (7d-CIDR) before NS (SynNS). Level 2: SynNS versus fixed-time artificial insemination (SynAI; 7d-CO-Synch + CIDR) with conventional semen. Level 3: conventional versus sex-skewed semen (FTAI-con and FTAI-sexed, respectively). Artificial insemination occurred 60–66 h post CIDR removal (SynAI; FTAI-con; FTAI-sexed) and bulls were introduced on d 0 (NS and SynNS) or 10–14 d post artificial insemination (SynAI; FTAI-con; FTAI-sexed). Breeding season PRs did not differ between treatments (*p* > 0.50). In Level 2, SynNS had greater 21 d PRs compared to SynAI (*p* < 0.01). For Level 3, FTAI-con had greater 21 d PRs compared to FTAI-sexed (*p* < 0.01). In Level 1, the proportion of cows that calved by day 14 was greater for SynNS compared to NS (*p* < 0.01). In Level 2, SynAI had a greater proportion calved by day 7 (*p* = 0.01); however, SynNS had a greater proportion calved by day 21 and 42 (*p* < 0.01). In Level 3, FTAI-con had a greater proportion calved by day 14 and 21 (*p* < 0.01) compared to FTAI-sexed. In conclusion, reproductive technologies altered the calving distribution with more calves born earlier.

## Simple Summary

Reproductive technologies are valuable tools for improving reproductive efficiency and management in beef cattle operations, but producers often hesitate to adopt them due to perceived complexity or inconsistent results. This study evaluated how different reproductive management levels influenced pregnancy rates and calving distribution in commercial beef herds over two years. Producers implemented stepwise reproductive strategies, beginning with natural service, progressing to estrus synchronization before natural service, then to fixed-time artificial insemination (AI) using conventional semen, and finally to AI with sexed semen. While overall pregnancy rates were similar among treatments, reproductive technologies affected the timing of calving. Estrus synchronization and AI protocols resulted in a greater proportion of calves born earlier in the calving season, which can increase calf weaning weights and improve management efficiency. Minor reductions in reproductive performance were observed during the first year of technology adoption, but these effects diminished in the second year as herds adapted to the new protocols. These findings emphasize that reproductive technologies can effectively improve herd reproductive performance and productivity over time, and that temporary setbacks should not discourage producers from implementing practices that provide long-term benefits to their operations.

## 1. Introduction

The adoption of reproductive technologies by beef producers in the US is low, with only 37.5% of all beef operations using any kind of reproductive technology [1]. Overall, the main method of breeding beef females is by natural service (NS) with 90.7% of beef operations using this as their only method of breeding [1]. Further, only 7.3% and 11.6% of beef operations use estrous synchronization and artificial insemination (AI), respectively [1]. According to USDA census data collected in 2007–2008 [2], lack of labor and time was the number one reason for producers not to adopt estrous synchronization and AI (39% and 38%, respectively), followed by cost (17% and 21%, respectively), and complexity (17% and 16%, respectively). The use of reproductive technologies, specifically estrous synchronization and AI, has the potential to improve genetic merit, calf uniformity, and weaning weight [3,4,5], thus increasing the profit potential for each calf.

Previous research has demonstrated that heifers that calve early (first 21 d of the calving season) in their first calving season remain in the herd longer and wean more pounds of calf through their sixth calf compared to heifers that calve later in their first calving season [6]. Furthermore, it has been reported that heifer calves born early in the calving season had increased pregnancy rates (PRs) and a greater proportion calved in the first 21 d of their first calving season [7,8]. Steer calves that were born early had increased weaning weight, final weight, hot carcass weight, and improved carcass quality compared to steers born later in the calving season; thus, steers born early had an increased value of approximately $25 to $74 per carcass [7,9]. The objectives of this study were to evaluate (1) the benefits of estrous synchronization with natural service; (2) the impact of AI compared to natural service following estrous synchronization; (3) the benefits of sexed semen compared to conventional semen; and (4) the impact of using reproductive technologies over multiple years in the same beef herds. It was hypothesized in Level 1 that females receiving estrous synchronization with natural service (SynNS) would have a shift in calving distribution with greater number of females calving earlier in the calving season compared to natural service (NS) only. In Level 2, it was hypothesized that no difference would be expected between SynNS and the use of fixed-time AI (SynAI). Lastly, in Level 3, it was hypothesized that use of sex-skewed semen (FTAI-sexed) would cause a delay in calving due to lower pregnancy rates at first service compared to conventional semen (FTAI-con); however, final pregnancy rates would not differ due to an increase in pregnancies generated from the second service (first cycle of the clean-up bull).

## 2. Materials and Methods

All procedures were approved by the South Dakota State University Institutional Animal Care and Use Committee protocol 15-109E.

### 2.1. Experimental Design

This study was divided into three levels of reproductive management. Producers started at either Level 1 or 2 based on the current reproductive management strategies of the operation. In the second year of the study, producers that were enrolled in Level 1 (*n* = 6 operations) were moved to Level 2 and producers enrolled in Level 2 (*n* = 5 operations) were moved to Level 3. One producer from Level 1 did not participate in year two. The number of females exposed per level per herd is shown in Table 1. All females used in this study were Bos taurus, specifically Angus or Angus crosses.

Within each herd, cattle were divided into two treatments per level. In year 1, females were sorted by age, days postpartum, and body condition score. In year 2, cows were blocked based on previous treatment and sorted by age, days postpartum, and body condition. Level 1 included females exposed to natural service only (NS) or exposed to NS following an estrous synchronization protocol (SynNS). Females in the SynNS group received a CIDR (Zoetis, Kalamazoo, MI, USA) insert 7 d prior to the start of the breeding season. The CIDR was removed on day 0 when females were exposed to bulls. Females in the NS group were exposed solely to bulls beginning on day 0. Both treatments were comingled and exposed to the same bulls in the same pastures. Animals in Level 2 were divided into (1) SynNS (as described in Level 1) and (2) artificially inseminated (AI-ed) following the 7 d CO-Synch + CIDR fixed-time AI protocol (SynAI). Bulls were turned out with the SynNS group on the day of CIDR removal, whereas cows in the SynAI group were AI-ed 60 to 66 h after CIDR removal and prostaglandin F2α injection. Clean-up bulls were turned out with SynAI cows 10 to 14 d after AI. Level 3 evaluated semen type with fixed-time AI (FTAI). Beef females were divided into (1) FTAI with conventional semen (FTAI-con) and (2) FTAI with sex-skewed semen (FTAI-sexed). Both groups were synchronized using the 7 d CO-Synch + CIDR protocol and AI-ed at 60 to 66 h post prostaglandin F2α injection, and clean-up bulls were turned out with cows 10 to 14 d after AI. Breeding season was approximately 60 days long starting at bull turnout (NS and SynNS) or the first AI (SynAI, FTAI-con, and FTAI-sexed). All bulls were required to pass a breeding soundness exam conducted by a licensed veterinarian prior to the start of each breeding season and bull-to-cow ratio was kept at 1 bull to 30 cows or less to avoid insufficient bull power. A serving capacity test was not performed on bulls prior to the breeding season. In the following calving season, calf birth date was recorded, and the calving distribution (CD) was calculated. The day that the third calf born alive was recorded and considered day 1 of the calving season.

### 2.2. Pregnancy Rates

Pregnancy status was determined at >30 d after the end of the breeding season by transrectal ultrasonography performed by experienced technicians. Females were considered pregnant when a viable embryo or fetus (presence of a heartbeat or movement) was identified. Transrectal ultrasonography was also used to determine fetal age [i.e., AI and “early bull” (conceived within the first 21 d of the breeding season) pregnancies] by measuring the fetus crown–rump length, trunk diameter, or eye orbital according to E.I. Medical, Ivos II ultrasound machine settings. Calf genetic parentage was not tested.

### 2.3. Economics

For the economic analysis, it was assumed that all calves were sold at weaning. Additional assumptions included that calves born on day 1 of the calving season weighed 700 lbs (318.2 kg) at weaning (regardless of sex) and that calves gained 1 kg per day. Therefore, for each day a calf was born later in the calving season, 1 kg was deducted from its weaning weight compared with calves born on day 1. A rolling price was not considered; instead, an average price of $400/cwt was used to calculate revenue. The average value per animal was calculated for each treatment within the herd, reported as mean ± SD. The difference in per-calf value between technologies was determined by subtracting the value of the least technological management from that of the most technological management.

### 2.4. Statistical Methods

All data were evaluated using SAS (9.4) software. The GLIMMIX procedure was used to compare the effect of treatments within each Level (1, 2, and 3) on final breeding season PRs, the percentage of early pregnancies (conceived in the first 21 d of the breeding season), and the proportion of cows that calved by d 7, 14, 21 and 42 of the calving season. The mean and median calving day was calculated for each producer and treatment and evaluated with the MIXED procedure in SAS. Producer was used as a random effect for all models; year was included as a fixed effect only for Level 2 analyses because Levels 1 and 3 included only one year of observation. Calving distribution was evaluated with the LIFETEST (Wilcoxon test) procedure in SAS to compare treatments within each Level. Also, the LIFETEST procedure was used to compare calving distribution between technologies used for the first time versus the second year within treatment for SynNS and SynAI (FTAI-con). The GLIMMIX procedure was used to compare the proportion of cows that calved by d 7, 14, 21 and 42 d of the calving season.

## 3. Results

**Figure 1:**
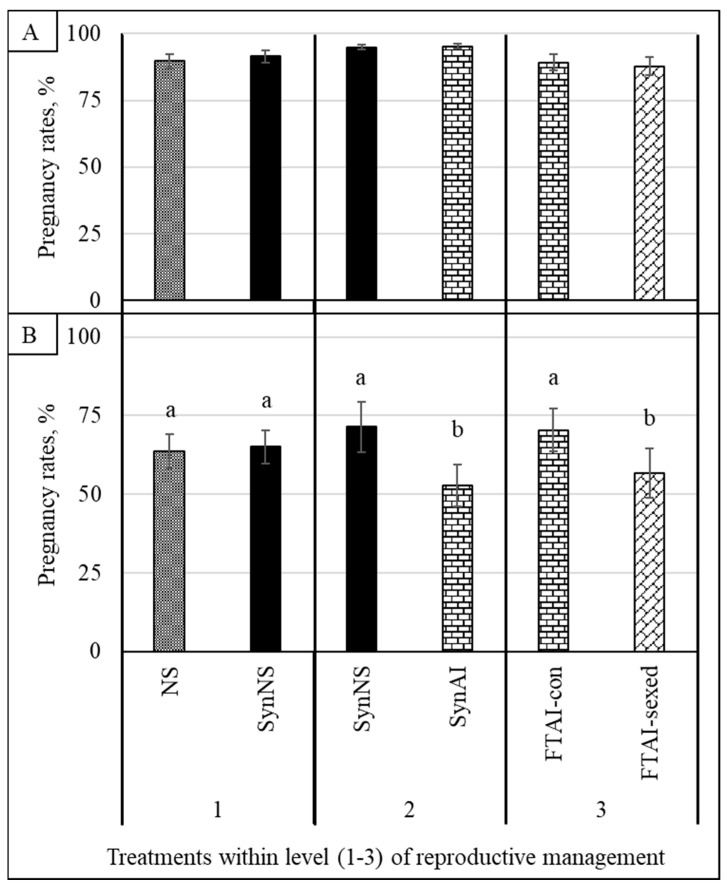
Overall pregnancy rates (panel (**A**)) and percentage of females pregnant in the first 21 d of the breeding season (panel (**B**)) per treatment by Level (1, 2 or 3) of reproductive management. In panel (**A**), there was no difference between treatments within each level of reproductive management (*p* > 0.50). In panel (**B**), there was no difference in Level 1 (*p* = 0.76); however, there was a difference in Levels 2 and 3 (*p* < 0.001). NS = natural service; SynNS = synchronized and bred by natural service; SynAI = synchronized and fixed-time artificial insemination (FTAI); FTAI-con = synchronized and FTAI with conventional semen; FTAI-sexed = synchronized and FTAI with sex-skewed semen. ^a,b^ Bars not sharing a common superscript within a level differ (*p* < 0.001).

Breeding season PRs did not differ between treatments within each Level (*p* > 0.50; Figure 1A). There was no difference (*p* = 0.76; Figure 1B) in 21 d PRs (early pregnancy) for Level 1; however, SynNS had greater 21 d PRs compared to SynAI (Level 2), and FTAI-con had greater 21 d PRs compared to FTAI-sexed (Level 3; *p* < 0.001; Figure 1B).

There was no difference in mean calving day for Level 1 (NS = 21.3 ± 4.1; SynNS = 19.7 ± 4.1; *p* = 0.56). There was, however, a difference in mean calving day for Level 2 (19.8 ± 2.4 vs. 24.0 ± 2.4 for SynNS and SynAI, respectively; *p* = 0.03). Level 3 tended (*p* = 0.09) to differ in mean calving day between FTAI-con and FTAI-sexed (17.6 ± 1.9 vs. 21.7 ± 1.9, respectively). Median calving day was not different within Level 1 (NS = 17.9 ± 4.3; SynNS = 17.0 ± 4.3; *p* = 0.73) or within Level 2 (SynNS = 16.2 ± 2.8; SynAI = 20.1 ± 2.8; *p* = 0.18); however, within Level 3 median calving day tended (*p* = 0.09) to differ between FTAI-con (12.7 ± 2.4) and FTAI-sexed (20.4 ± 2.4).

**Figure 2:**
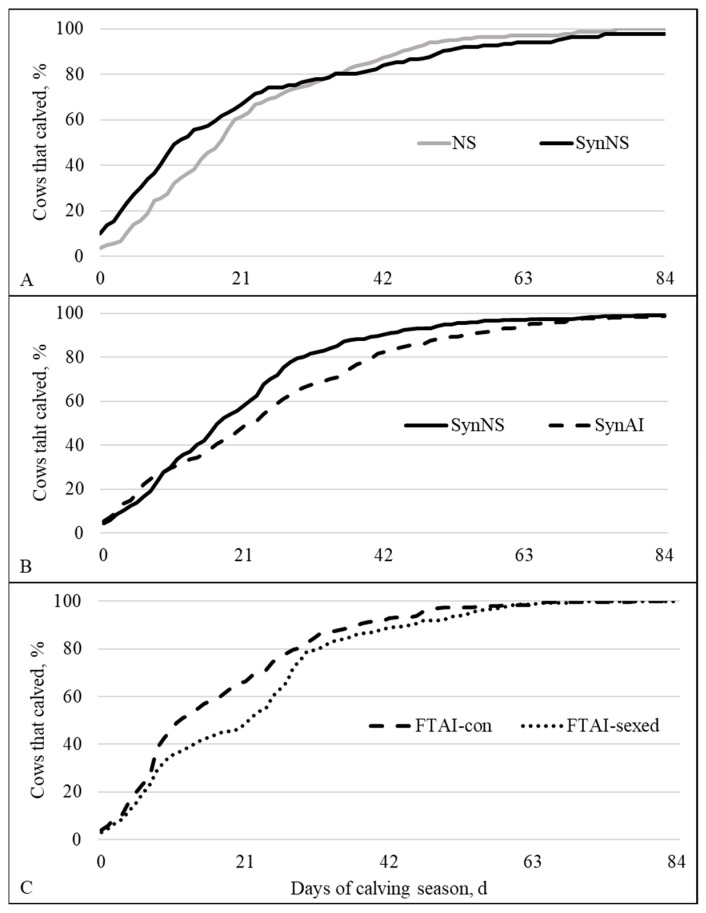
Calving distribution for Levels 1, 2, and 3 (panels (**A**), (**B**), and (**C**), respectively). There was a difference (Wilcoxon *p* < 0.02) in calving distribution between treatment for all levels. NS = natural service; SynNS = synchronized and bred by natural service; SynAI = synchronized and fixed-time artificial insemination (FTAI); FTAI-con = synchronized and FTAI with conventional semen; FTAI-sexed = synchronized and FTAI with sex-skewed semen.

**Figure 3:**
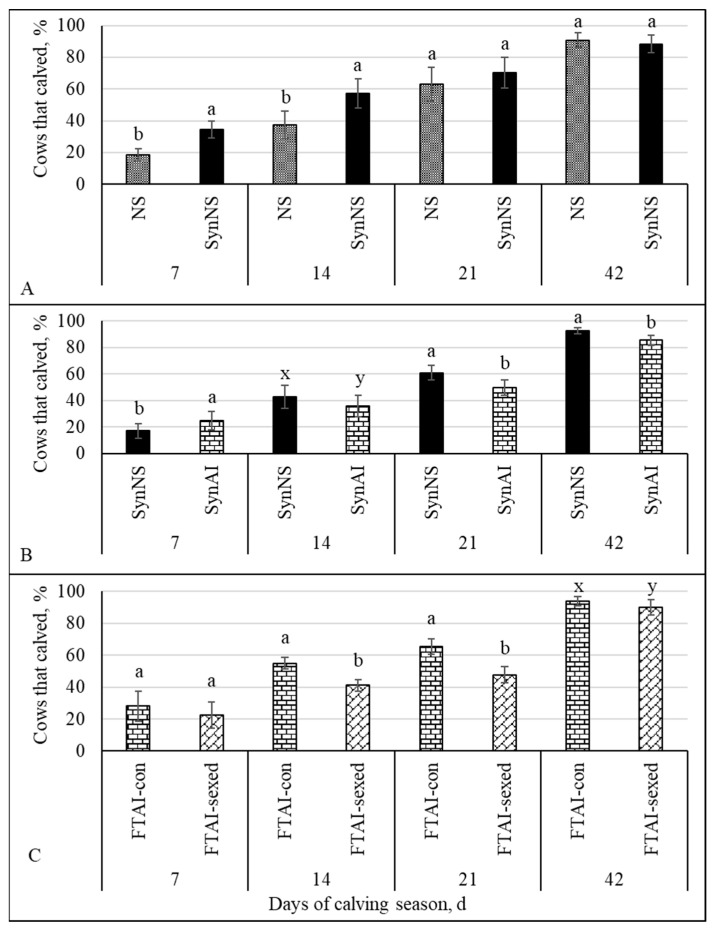
Cumulative proportion of females that have calved for Levels 1, 2 and 3 (panel (**A**), (**B**), and (**C**), respectively) for each treatment on the first 7, 14, 21, and 42 d of the calving season. NS = natural service; SynNS = synchronized and bred by natural service; SynAI = synchronized and fixed-time artificial insemination (FTAI); FTAI-con = synchronized and FTAI with conventional semen; FTAI-sexed = synchronized and FTAI with sex-skewed semen. ^a,b^ Bars not sharing a common superscript within a day differed (*p* ≤ 0.01). ^x,y^ Bars not sharing a common superscript within a day differed (*p* = 0.09).

Calving distribution was affected by treatment at all levels (Wilcoxon test *p* < 0.02; Figure 2). In Level 1, the cumulative proportion of cows that calved in the first 7 and 14 d was greater for SynNS compared to NS (*p* < 0.01); however, there were no differences at day 21 or 42 (*p* ≥ 0.21; Figure 3A). In Level 2, SynAI had a greater cumulative proportion of females that calved by day 7 (*p* = 0.01) compared to SynNS; however, SynNS tended to have a greater proportion that calved by day 14 (*p* = 0.09) compared to SynAI. At day 21 and 42 SynNS had a greater cumulative proportion of females that had calved (*p* < 0.005; Figure 3B) compared to SynAI. In Level 3, there was no difference in calving rate between treatments in the first 7 d (*p* = 0.16; Figure 3C). Nevertheless, by days 14 and 21 FTAI-con had a greater proportion of females that had calved (*p* ≤ 0.001), and by day 42 it tended (*p* = 0.09) to have a greater proportion of females that had calved compared to FTAI-sexed (Figure 3C).

**Figure 4:**
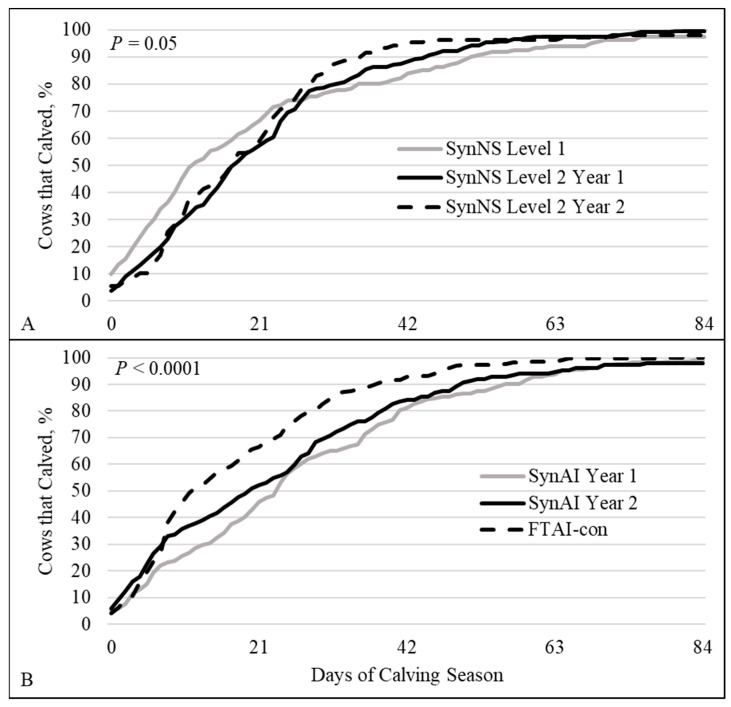
Calving distribution for SynNS (panel (**A**)), and SynAI and FTAI-con (panel (**B**)). There was a difference (Wilcoxon *p* ≤ 0.05) in calving distribution between first time using the technology compared to second time using the technology. SynNS = synchronized and bred by natural service; SynAI = synchronized and fixed-time artificial insemination (FTAI) with conventional semen; FTAI-con = synchronized and FTAI with conventional semen.

The length of reproductive technology use impacted CD (Figure 4). In the second year using SynNS (SynNS Level 2 Year 2), >95% of cows had calved by day 42 of the calving season in comparison to the first year when it took 68 d and 53 d (Level 1 and Level 2, respectively) to reach >95% of cows calving (Figure 4A). During year 1, the SynNS Level 1 had a greater (*p* ≤ 0.04) percentage of cows that calved within the first 7 (34.0 ± 3.7) and 14 (55.6 ± 3.9) d of the calving season compared to SynNS Level 2 (d 7 = 20.0 ± 2.6; d 14 = 39.2 ± 3.2).

In terms of estrous synchronization and AI, the separation between first-time use of FTAI and the second year of use was clearly distinguished. Level 2 (first year using FTAI) had similar CD (both years of the study) while FTAI-con (second year using FTAI) had a shift in calving distribution to earlier calving (Figure 4B). Over 95% of the FTAI-con cows calved by day 47; in comparison, SynAI (years 1 and 2) cows reached the same percentage on day 64 of the calving season (Figure 4B). There was no difference in the percentage of cows that calved in the first 7 d (*p* = 0.27) of the calving season; however, by day 14 FTAI-con had a greater (*p* < 0.01) percentage of cows that had calved (54.7 ± 3.1) compared to both SynAI years (30.4 ± 3.1, 40.4 ± 4.0, respectively). Also, SynAI in year 2 was greater than SynAI in year 1 (*p* = 0.05). On days 21 (66.4 ± 2.9; 46.0 ± 3.3; 52.3 ± 4.1) and 42 (92.8 ± 1.6; 81.3 ± 2.6; 84.1 ± 3.0) of the calving season FTAI-con continued to have a greater percentage of cows that had calved compared to SynAI in both year 1 and 2, respectively (*p* ≤ 0.006).

Change in revenue associated with treatment and year is reported in Table 2. There was a $7.77 increase in calf value when SynNS was compared to NS. When Syn AI was compared to NS and SynNS, there was a decrease in calf value of $3.30 and $11.07, respectively. Further, when SynSexed was compared to NS and SynAI, there was an increase in calf value of $1.56 and $4.86, respectively; however, there was a decrease of $6.21 in calf value when compared to SynNS.

## 4. Discussion

The adoption of technologies is paramount for an economical and sustainable beef operation. To better utilize resources available in an operation, a well-defined, short breeding season (60 to 90 d) is necessary. It is customary in cow–calf operations to wean all calves on one day (or a few days in large herds). Calves gain approximately 1 kg of body weight per day from birth to weaning; thus, calves born earlier in the calving season will be older and consequently heavier at weaning [6,7]. Furthermore, several studies have demonstrated the advantage of heifers born earlier in the calving season being more fertile than heifers born later [6,7,10]. Also, heifers that calved earlier in their first calving season remained in the herd longer, produced on average one extra calf, and weaned heavier calves through their sixth calf compared to heifers that calved late in their first calving season [6]. To further reiterate the importance of reproductive efficiency for an operation’s economic success, when a cow misses a pregnancy, the net present value (NPV) for her productive life is reported to be negative [11]. A recent study demonstrated that it took a cow 6.14 ± 2.88 (mean ± SD) calves to pay back her developmental period if she calved every year starting at 2 years of age; however, if a cow missed one calf, the payback period was increased to 8.18 ± 2.54 calves, and if she missed two calves, it increased to 9.61 ± 1.91 [12]. Thus, reproductive management and technologies that can increase the likelihood of females calving early as heifers and during their productive life have the potential to improve beef cattle profitability and sustainability.

One of the factors that influence pregnancy rates is cycling status at the beginning of the breeding season [13]. Longer days postpartum at the beginning of the breeding season increased the proportion of animals cycling, as the interval from parturition to first estrus can vary from 30 to 130 days in beef cows [14]. Stevenson et al. [15] reported that only 9% of suckled cows had returned to cyclicity 30 or less days postpartum, while 70% of cows with 70 or more days postpartum were cyclic. Although cycling status was not determined in the present study, days postpartum were accounted for when treatments were allocated. Since the number of days postpartum equally allocated to each treatment within each herd, it was not included in the statistical analyses. Also, it was reported that regardless of parity, females that calved early (within the first 22 days of the calving season) had greater cyclicity compared to cows that calved later [15]. Females that were cycling at the beginning of the breeding season had greater pregnancy rates compared to cows that were in anestrus [16,17].

In the current study, body condition score (BCS), days postpartum, and parity were not included in the model. Stevenson et al. [18] reported that cows with BCS greater than 5, more than 72 days postpartum at AI, and two or more calving events had higher pregnancy rates compared to cows with ≤5 BCS, ≤72 days postpartum, and those that were primiparous. Larson et al. [19] reported that for every unit increase in BCS from 3 to 6.5 there was an increase in the proportion of cow cycling of 11.9% (regardless of days postpartum), and an increase in pregnancy rates of 9.7%. Pregnancy rates were higher for cows with more than 50 days postpartum compared to those with fewer than 50 days postpartum [19]. Further, a recent study demonstrated a relationship between BCS and odds of early conceiving, where cows with BCS 3 and 4 had lower odds of becoming pregnant than cows with BCS between 5 and 7; however, it failed to demonstrate the relationship between the odds of pregnancy outcomes at final pregnancy diagnosis with BCS, age, or calving interval [20].

The main objective of this study was to provide science-based alternatives for beef producers to improve reproductive management using currently available technologies that could improve the efficiency, profitability and sustainability of the beef industry. The aim of this study was to compare different applications of reproductive technologies under similar management conditions in a pairwise comparison. Since the majority of the beef industry still relies on NS [1], Level 1 compared two methods of NS, with and without estrous synchronization. The inclusion of AI can benefit beef producers because it allows the use of animals with proven superior genetic merit and can hasten genetic improvement without the cost of purchasing higher-genetic-merit bulls. In addition, estrus synchronization with FTAI has been reported to shorten the calving season, increase calf uniformity, and increase pounds of calf produced per cow exposed [4,21,22]. Thus, estrous synchronization with or without AI was utilized in Level 2. Further, controlling the sex of the calf born can offer additional alternatives in herd management. In the dairy industry, female calves have greater value than male calves since the purpose of the industry is to produce milk and male dairy calves have lower market value [23]. Nevertheless, in beef industry, the difference in value between males and females is not as drastic, since females are necessary as breeding stock replacements, while males (particularly steers) have a greater market value. Thus, different breeding strategies can be performed using sex-skewed semen, such as increasing the number of males (steer) produced with improved terminal characteristics or producing replacement females from genetically superior sires while using terminal sires for the remaining of the herd. So, in Level 3, cows were AI-ed with conventional or sex-skewed semen. Even though this study was only conducted for two years, it gives a within-herd comparison of whether each treatment (Level 1 synchronization; Level 2 artificial insemination, and Level 3 sex-skewed semen) was beneficial or detrimental to reproductive performance.

In this study, it was reported that the use of reproductive technology, either estrous synchronization with natural service or FTAI, positively changed CD, while the positive changes were more apparent in the second year using the same technology (SynNS and FTAI). The use of CIDR in synchronization protocols for AI has been reported to improve pregnancy rates [18]; the use of a CIDR has the potential to increase the number of cycling females during the breeding season as it has been reported to induce cyclicity in anestrus postpartum beef cows [24]. Thus, it is possible to speculate that some of the positive changes in CD observed in the current study may be attributed to anestrus cows returning to cyclicity earlier due to progestin exposure from the CIDR. Since the study was designed to compare within-level effects, year-to-year variation should be interpreted with caution; nevertheless, similar results were reported by Mercadante et al. [3], where reproductive performance and economics of a beef herd were improved as reproductive technologies were introduced and continued to improve over the years over the duration of the study. In addition, reports from Brazil demonstrated that pregnancy per AI increased from 2007 to 2015 for heifers (from 39.6% to 48.5%), primiparous cows (from 44.5% to 47.1%), and multiparous cows (from 49.7% to 54.1%); the number of females represented increased almost exponentially, from 30,805 in 2007 to 597,888 in 2015 [5]. Although physiological changes within the herd, such as improvement of days postpartum by the beginning of the breeding season may have had an effect, adaptation of within-herd management is still a great source of improvement in herds adopting reproductive technologies. In the present study, when SynNS was compared to SynAI, there was a delay in calving for SynAI that could be attributed to clean-up bulls not being introduced to the group until 10 to 14 d after AI. This was implemented intentionally to allow ultrasound technicians to differentiate between AI and NS pregnancies. There could also be variation in gestation intervals between AI and natural service sires, as calves were not tested for parentage and were classified based on ultrasound age and calving date. Additionally, since SynAI represents the first year that producers were utilizing AI, it is possible that the introduction of the new management may have caused the differences. Nevertheless, taking into consideration mean calving day, NS results (NS and SynNS) ranged from 21.3 to 19.7 d, whereas in the first year of using FTAI (SynAI) it was 24.0 d, and in the second year (FTAI-con) it dropped to 17.6 d. In a previous study comparing FTAI to NS only (no estrous synchronization), the authors reported an improvement in mean calving day from 31.3 to 26.8 when FTAI was implemented [4]. Further, when comparing median calving day, or the number of days required to reach 50% of cows to calve, groups using NS had median values ranging from 16.2 to 17.9 d; however, SynAI had a median of 20.1 d and FTAI-con had a median of 12.7 d. Lastly, in the second year using estrus synchronization, regardless of AI or NS, the number of days required to reach the end of the calving season (>95% of cows calving) decreased. These results are consistent with previously reported changes in calving distribution, showing that the longer AI was utilized, the earlier cows calved during a 6-year observation period, with a great concentration of females calving within the first 21 d of the calving season [3]. In comparison, the reported mean calving day (17.6 to 24.0) for the current study regardless of technology was earlier than that reported previously [4]. This highlights that herds used in the current study already had a well-defined short breeding and calving season with left-skewed calving distribution, as desired by the beef industry, prior to the initiation of the research. According to the latest USDA report, 58.7% of beef operations do not have a defined breeding season [1]. Because of the left-skewed calving rate even within the NS group, it would be expected that the mean number of days postpartum in these farms was high at the beginning of the breeding season. As previously reported, increased days postpartum are associated with a greater likelihood of females resuming cycling at the onset of the breeding season, and cycling females have been reported to have greater pregnancy rates [15,16,17]. In herds without a defined breeding season, it is likely that a greater proportion of females would have a lower number of days postpartum at the beginning of the breeding season, resulting in greater numbers of anestrus females. Anestrus is the greatest factor impacting cow fertility [13,25]. The use of progesterone (and analogs) for a short period of time (5 to 9 days) has been reported to induce cyclicity in anestrus cows [24,26]. Thus, the positive impact of reproductive technologies in herds where a controlled breeding season is not adopted may be even greater compared to the results reported in the present study.

A study conducted at Colorado State University indicated that cows that conceived during a synchronized estrus calved on average 13 d earlier than cows that were not synchronized [27]. Lamb et al. [28] reported that cows synchronized with a 7 d CIDR had a higher pregnancy rate (43%) in the first 10 days of the breeding season compared to cows that were not synchronized (35%), using a ratio of one bull to up to 30 females; however, pregnancy rates were similar by day 30 (64.4% vs. 64.7%, respectively), and at the end of the breeding season (89.7% vs. 89.6%, respectively). Further, the success of SynNS is dependent on the bull-to-female ratio as bulls exposed to synchronized cows will have more females to be bred in a shorter period of time compared to non-synchronized females. For non-synchronized females, the ratio can be one bull to 10 to 60 cows, depending on age, experience, and semen quality of the bull. In synchronized groups of females, the recommended rate is one bull to up to 25 females. Healy et al. [29] reported that pregnancy rates over a 28-day synchronized breeding season tended to be reduced when a bull-to-female ratio of 1:50 (77%) was used compared to a bull-to-female ratio of 1:16 (84%); however, no difference was detected between a bull-to-female ratio of 1:16 and 1:25 (83%). In a recent retrospective study, the bull-to-cow ratio was investigated for 392 breeding groups, with 14,868 females represented [30]. In that study, all cows received one round of FTAI with bull exposure 10 days later for a total breeding season of 70 d. Breeding groups were either single- or multiple-sire pastures, with bull-to-cow ratio ranging from 1:9 to 1:73. When considering only cows not pregnant to FTAI, the ratio ranged from 1:2 to 1:44. A negative correlation between bull-to-open cow ratio was reported; however, it only explained 1% to 11% of variation in pregnancy rates observed. Further, no difference in pregnancy rates due to bull-to-cow ratio was reported when the ratio was separated into quartiles based on total cows exposed or only open cows exposed at first estrus after FTAI and during subsequent estrus. Thus, the authors concluded that the bull-to-cow ration was not the main limiting factor even when a ratio higher than 1:30 was used [30]. Although return to estrus was not synchronized in the aforementioned study, it is expected that non-pregnant females submitted to the FTAI protocol will return to estrus within a 3 d window [19]. Considering that the maximum number of females reported in estrus on one day was 18% [19], it would be expected that, for a bull to mate with 5 to 6 cows in one day and assuming a 3 d period of peak return to estrus activity (47%; [19]), a bull would have to mate with 14 cows in a 1:30 bull-to-cow ratio. Thus, it is possible to assume that the use of a 1:30 bull-to-cow ratio in the present study was not a limiting factor for pregnancy rates [30].

In this study, the use of sex-skewed semen was not advantageous to reproductive efficiency, because it decreased the proportion of cows that conceived early and calved early, and tended to decrease the proportion of cows calving by day 42 of the calving season. In addition, using sex-skewed semen tended to increase the mean and median calving day. Further research, especially evaluating the long-term impact, is needed to identify the best strategy to incorporate sex-skewed semen within commercial beef cattle operations where the difference in calving distribution is offset by the change in sex ratio and increased value of the selected calf sex. Sex-skewed semen was commercially available in 2003, and early reports in dairy herds reported similar results with sex-skewed semen generating approximately 80% of conventional semen in Holstein heifers [31]. Recently, studies have demonstrated differences in PRs between females that exhibited behavioral estrus prior to FTAI and those that did not exhibit behavioral estrus [32,33]. Previous studies have reported a reduction in PRs when using sex-skewed semen compared to conventional semen [34,35,36]. When the expression of behavioral estrus was evaluated, sex-skewed semen caused a small decrease in comparison to conventional semen; however, that difference in PRs was greater for females that did not exhibit behavioral estrus [34]. In the present experiment, all females assigned to the FTAI-sex group were inseminated with sex-skewed semen regardless of estrus at the time of AI. Thus, reduction in pregnancy rates and delay in calving could be minimized by using conventional semen in females that have not expressed behavioral estrus by the time of AI.

In the current dataset, the use of sexed semen increased per animal revenue compared with both NS and conventional semen; however, it did not increase revenue when compared with SynNS. Interestingly, on average, the use of FTAI with conventional semen did not increase revenue. Nevertheless, when examining Level 3 results, the value of calves generated through AI with conventional semen was the greatest across all levels and years (ranging from $23.92 to $81.09), further highlighting the long-term economic impact of reproductive technologies on herd performance. This is in accordance with what has been previously reported by Mercadante et al. [3] where an increase in calf value was observed across the years of the study. This economic analysis considered only the revenue generated from selling all calves. Several factors that could influence animal performance and, consequently, revenue were not included in this analysis. For example, AI-sired calves may exhibit greater growth potential due to the use of higher-genetic-merit sires, and the use of sexed semen skews calf sex ratios. Additionally, this analysis did not account for the costs associated with each technology and the number of bulls needed for each technology; therefore, profitability was not assessed.

When reproductive technologies are applied in a production setting it is important to evaluate the current status of the herd and the experience of the management. The results of this study could vary greatly depending on the status of the herd before implementing any technology. If a herd did not previously have a defined breeding season, the benefits of synchronization could differ greatly as cows would not be far enough postpartum to be cycling. The CD in present study was already tightly clustered early in the season, which likely limited detectable shifts in CD with synchronization; additionally, delayed bull turnout in AI treatments (10–14 d) may have reduced early calving responses, although it was necessary for accurate pregnancy diagnosis. Thus, it is important to evaluate the herd before implementing any changes in management.

## 5. Conclusions

Reproductive technologies can improve calving distribution; however, minor setbacks in calving distribution may happen in the first year a reproductive technology is introduced. Nevertheless, in the second year, the setbacks were overcome under the conditions of this experiment, which involved a combination of the cows’ physiological status in year 2 and improved herd management. Beef producers need to be prepared for a possible short-term decrease in reproductive efficiency when considering the adoption of reproductive technologies so that they do not abandon the practice before the benefits are realized. Further research in the use of sex-skewed semen in commercial beef cattle operations is warranted to minimize reductions in reproductive efficiency.

## Figures and Tables

**Table 1:** Number of females enrolled by producer at each level of reproductive management participation.

Producer	Level		
	1	2	3
1	104	87	-
2	108	74	-
3	96	76	-
4	35	36	-
5	64	62	-
6	31	-	-
7	-	246	207
8	-	97	121
9	-	70	45
10	-	105	220
11	-	92	134
Total	438	945	727

**Table 2:** Average revenue value in US dollars (mean ± SD) per treatment and year for calves born from cows in the treatments: natural service only (NS), estrous synchronization with natural service (SynNS), estrous synchronization and artificial insemination with conventional semen (SynAI), estrous synchronization and artificial insemination with sex-skewed semen (FTAI-sexed). The average price used was $400/cwt regardless of weight and sex of the calf.

Year	Level	NS	SynNS	SynAI	FTAI-Sexed	Average Level
1	1	2608.11 ± 146.19	2616.67 ± 202.97			2612.35 ± 176.67
AVG1&2	2		2615.51 ± 147.93	2575.17 ± 182.41		2594.5 ± 167.99
1	2		2612.3 ± 147.45	2565.66 ± 175.21		2589.79 ± 163.13
2	2		2622.83 ± 148.75	2589.27 ± 191.71		2603.03 ± 176.14
2	3			2646.75 ± 128.50	2609.66 ± 139.67	2627.86 ± 135.58
	Average Treatment	2608.11 ± 146.19	2615.88 ± 167.49	2604.81 ± 166.06	2609.66 ± 139.67	2609.53 ± 160.34

## Data Availability

The raw data supporting the conclusions of this article will be made available by the authors on request.

## Abbreviations

The following abbreviations are used in this manuscript:

AI	Artificial Insemination
FTAI	Fixed-Time Artificial Insemination
PR	Pregnancy Rate
NS	Natural Service
SynNS	Estrous Synchronization and Natural Service—Treatment Name
SynAI	Estrus Synchronization with Fixed-Time Artificial Insemination with Conventional Semen—Treatment Name
FTAI-con	Estrus Synchronization with Fixed-Time Artificial Insemination with Conventional Semen—Treatment Name
FTAI-sex	Estrus Synchronization with Fixed-Time Artificial Insemination with Sex-Skewed Semen—Treatment Name
CD	Calving Distribution
